# A tale of two sisters: identical *IL36RN* mutations and discordant phenotypes

**DOI:** 10.1111/bjd.14003

**Published:** 2015-11-25

**Authors:** N. Rajan, N. Sinclair, H. Nakai, Y. Shimomura, S. Natarajan

**Affiliations:** ^1^ Institute of Genetic MedicineNewcastle UniversityNewcastle upon TyneNE1 3BZU.K; ^2^ Graduate School of Science and TechnologyNiigata UniversityNiigataJapan; ^3^ Laboratory of Genetic Skin DiseasesNiigata University Graduate School of Medical and Dental SciencesNiigataJapan; ^4^ The James Cook University HospitalMiddlesbroughTS4 3BWU.K

dear editor, Recessive mutations in *IL36RN*, encoding interleukin 36 receptor antagonist (IL‐36Ra), have illuminated the understanding of the pathogenesis of generalized pustular psoriasis (GPP).^1^, ^2^ Consanguineous Tunisian families were central to the discovery of homozygous mutations in *IL36RN* being associated with early‐onset GPP.^2^ Studies in populations from different genetic backgrounds highlighted the requirement of other genes in cases that are mutation negative or that only carry a mutation in a single allele.^3^ Sibling pairs with compound heterozygote mutations in European and Asian populations, an intriguing, nonconsanguineous, genetic background to study phenotypes associated with *IL36RN*, are rare. Furthermore, the genetic differences in autozygosity seen in consanguineous families are currently being delineated at a population level, and support the exploration and comparison of phenotypes seen in this genetic background with other nonconsanguineous genetic backgrounds.^4^ Only one compound heterozygous case of Japanese twins has been reported as affected siblings. In this case, both twins developed GPP at the age of 2 years, following treatment with amoxicillin. Siblings with homozygous *IL36RN* mutations have been reported to present within 11 years of each other (Table S1; see Supporting Information). Here, we present a report of two sisters that carry identical compound heterozygous mutations in *IL36RN*, yet first presented 34 years apart, highlighting the requirement of further genetic or environmental factors.

A 38‐year‐old European woman, with features consistent with severe GPP since 6 months of age, had been attending our department. Widespread small pustules were seen on the trunk and limbs, and numerous hospital admissions with severe pustulation and fever were a recurrent feature of childhood (Fig. 1a). The patient's history was negative for recognized triggers of GPP such as antibiotic usage, infection and menstruation. Histological examination of the sterile pustules demonstrated a brisk neutrophilic infiltrate, with some spongiosis (Fig. 1d). Acrodermatitis continua of Hallopeau involving the fingernails prevented nail growth. Extensive scalp involvement could not be controlled such that the patient resorted to wearing a hat. Pustules were seen on the oral mucosa during severe flares, and widespread lymphadenopathy was noted. Methotrexate therapy was commenced at the age of 6 years, and used alone and in combination with etretinate, but neither regime gave good disease control. Hydroxyurea was ineffective. Oral prednisolone suppressed pustulation but could not be sustained owing to Cushingoid side‐effects. Deterioration of her skin was noted during both her pregnancies, consistent with a diagnosis of impetigo herpetiformis, warranting treatment with ciclosporin during the second and third trimesters of her first pregnancy, and an elective termination of her second pregnancy. In addition to the physical morbidity the patient had been treated for depression relating to her cutaneous disease.

**Figure 1:**
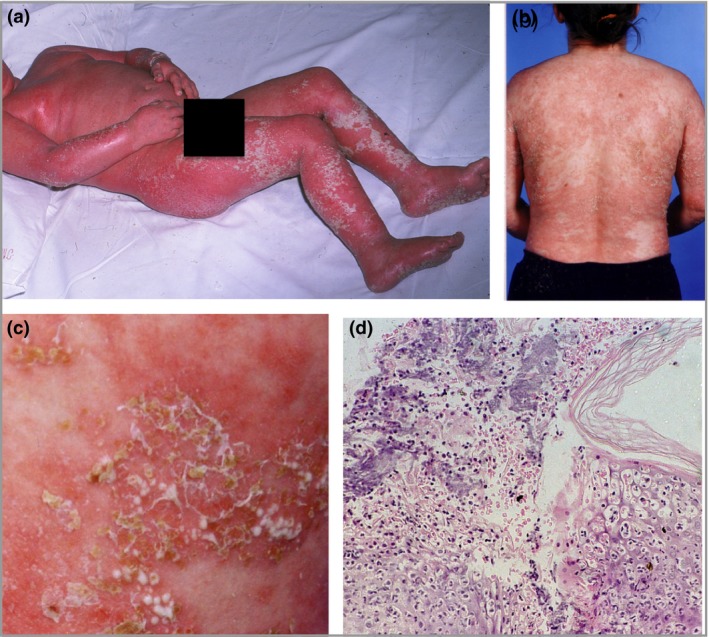
Clinical features and histopathological findings of the patient. (a) The proband at the age of 6 years, with generalized pustular lesions seen on the torso and legs, on a background of erythroderma. (b) The proband prior to starting antitumour necrosis factor therapy at the age of 30 years, with (c) a close‐up image highlighting the pustulation and superficial desquamation seen. (d) A dense neutrophilic infiltrate seen in the epidermis in a skin biopsy of a pustule taken at the age of 6 years.

Given the severity of her disease, recombinant soluble tumour necrosis factor‐α receptor treatment was started at the age of 30 years (Fig. 1b, c). Following treatment with etanercept at a dose of 25 mg subcutaneously (SC) twice weekly, her cutaneous disease activity was brought under control. Methotrexate and prednisolone were weaned successfully, and the patient experienced normal nail growth for the first time. After 11 months of treatment, she demonstrated plaque psoriasis without pustulation. The patient was then treated with adalimumab 40 mg SC every 2 weeks, with improved control, and has continued on this for the last 6 years. There have been no hospital admissions for the last 7 years.

By contrast, the proband's sister, who had never previously had skin disease, first developed widespread pustular psoriasis, warranting hospital admission, at the age of 34 years. Her clinical history was negative for antecedent infection, medication use or pregnancy. She was admitted to hospital and managed with topical therapy, prompting resolution of the rash after 12 days. She has subsequently had minimal disease affecting the scalp for a 6‐month period. Notably, she did not have oral pustules.

Mutation analysis of *IL36RN* was performed given the phenotype, and demonstrated identical mutations (sequencing primers and conditions as previously described).^1^ Both sisters carried compound heterozygous mutations in exon 5 of *IL36RN*: c.338C>T (p.Ser113Leu) and c.368C>T (p.Thr123Met) (Fig. 2a). The c.338C>T mutation is a known founder mutation in European populations, while c.368C>T has only been reported in Japanese cases,^5^ making this a novel finding in Europeans. As the mutation occurred at a CpG dinucleotide sequence, we postulate that the position c.368C of *IL36RN* could be a mutational hotspot across different populations. Polymerase chain reaction products were cloned to confirm that the two mutations resided on separate alleles.

**Figure 2:**
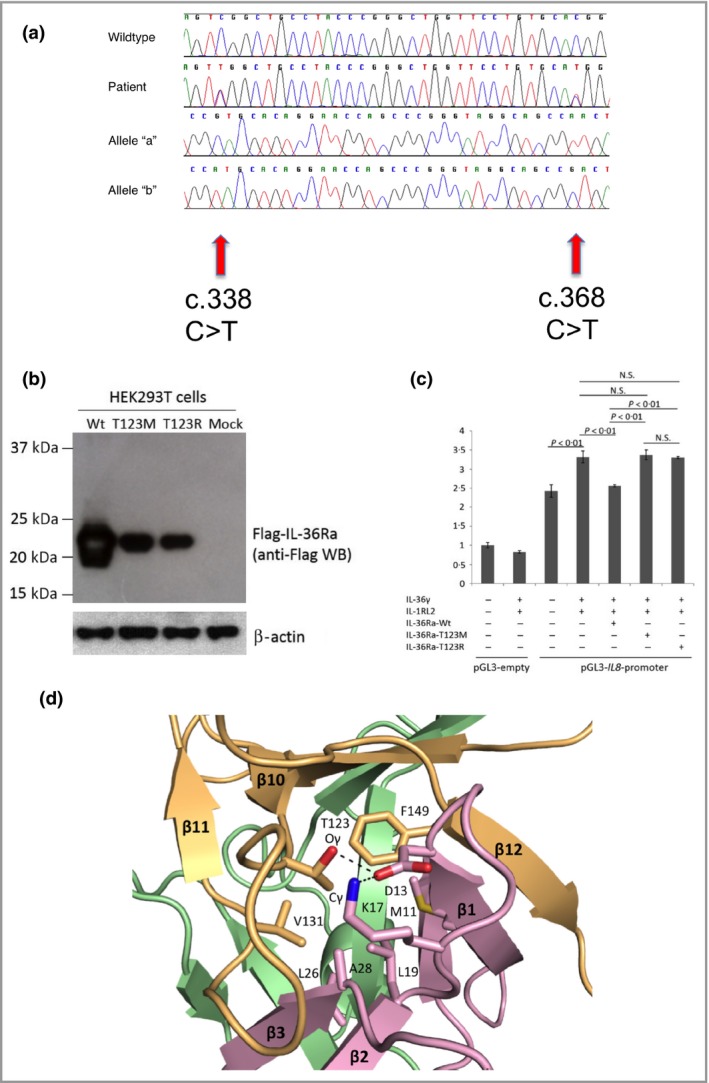
Mutation analysis of *IL36RN* in both patients, and functional and molecular evidence supporting the pathogenicity of the Thr123 (T123R) change. (a) Sanger sequencing analysis revealed compound heterozygous mutations in the proband's DNA. Mutations are shown at two positions within *IL36RN* exon5 (‘Patient’), c.338C>T and c.368C>T, which differs from the wild‐type sequence (‘Wild type’). Single allele analysis, carried out by cloning the polymerase chain reaction products followed by sequencing, reveals the presence of the c.338 C>T mutation (‘Allele ‘a’) and the c.368C>T on the other chromosome (‘Allele ‘b’). (b) Expression of the p.Thr123Met mutant interleukin 36 receptor antagonist (IL‐36Ra) protein is severely impaired compared with that of the wild type (Wt) IL‐36Ra in HEK293T cells. All constructs were verified by direct sequencing. β‐Actin was blotted as a normalization control. (c) *IL8* promoter–reporter gene assays demonstrate loss of function of the p.Thr123Met mutant IL‐36Ra protein in HeLa cells. The *IL8* promoter activity induced by interleukin (IL)‐36γ and IL‐1RL2 was significantly downregulated by the Wt IL‐36Ra (*P* < 0·01, Student's *t*‐test) but not by the p.Thr123Met or the p.Thr123Arg mutant IL‐36Ra. All experiments were performed in triplicate and repeated three times. The error bar indicates the SEM of a single representative experiment. NS, not significant. (d) Diagrams of the core hydrophobic patch in the human IL‐36Ra protein. The three‐dimensional structure of human IL‐36Ra was modelled (Swiss model; http://swissmodel.expasy.org/) using a reported murine IL‐1F5 structure (Protein Data Bank identifier: 1MD6) as a template.^8^, ^9^, ^10^ The predicted structure was generated using Pymol v0.99 (DeLano Scientific, San Carlos, CA, U.S.A.). Three β‐hairpins of IL‐36Ra are shown by purple (residues 1–46), green (residues 47–104) and orange (residues 105–155) ribbon representations. Amino acid residues that contribute to the stability of the conformation of IL‐36Ra, which interact with Thr123, are highlighted by stick presentations in red and blue. Suggested hydrogen bonds between these residues are shown as dotted lines.

In order to determine the biochemical effect of the missense mutation p.Thr123Met on the expression of IL‐36Ra, we generated an *N*‐terminal FLAG^®^‐tagged expression vector for the p.Thr123Met mutant (Met‐mutant) protein using the QuikChange^®^ site‐directed mutagenesis kit (Stratagene, La Jolla, CA, U.S.A.) with the wild‐type (Wt) IL‐36Ra expression vector as a template.^6^ A p.Thr123Arg‐Mut vector (Arg‐mutant), containing a mutation known to influence IL‐36Ra levels, was used as a positive control. Then, we overexpressed the Wt, Met‐mutant and Arg‐mutant FLAG^®^–IL‐36Ra vectors in HEK293T cells, collected the cell lysate and performed sodium dodecyl sulfate polyacrylamide gel electrophoretic separation of proteins followed by immunoblotting with mouse monoclonal anti‐FLAG^®^ M2 antibody (diluted 1: 1000; Sigma‐Aldrich, St Louis, MO, U.S.A.). Similar to the Arg‐mutant of IL‐36Ra protein, expression of the Met‐mutant was markedly reduced compared with the Wt, suggesting instability of the Met‐mutant IL‐36Ra protein (Fig. 2b).^6^

To investigate the functional effect of this instability, we performed reporter gene assays using the *IL8* promoter in HeLa cells, as described previously.^6^ The Wt IL‐36Ra protein significantly repressed the promoter activity induced by IL‐36γ and IL‐1RL2 (Fig. 2c), whereas neither the Met‐mutant nor the Arg‐mutant IL‐36Ra proteins reduced the activity (*P* < 0·01; Student's *t*‐test), consistent with the mutation resulting in loss of function (Fig. 2c).

To improve our understanding of how the mutation may have an effect on tertiary protein structure, we performed molecular modelling of the patient's mutation (Thr123‐>Met) (Fig. 2d). Thr123 is predicted to be situated in loop10 of IL‐36Ra, which is a structural element for a core hydrophobic patch, where a large number of amino acid residues are densely embedded. The mutation into methionine at Thr123, which has a bulkier side chain than threonine, is likely to cause steric clashing with adjacent amino acid residues. Furthermore, the mutation is predicted to disrupt the hydrophobic interaction and hydrogen bond network contributing to the stabilization of IL‐36Ra conformation, resulting in protein misfolding. Taken together with the overexpression and reporter studies, and previous clinical data of changes at this residue, these data support the finding that the c.368C>T mutation in our patient is pathogenic.

We present this case to highlight the strikingly distinct phenotypes seen in two *IL36RN* mutation carriers from the same nonconsanguineous pedigree. Homozygous carriers of previously identified *IL36RN* mutations across families have been observed to have a range of age of presentations, which suggests that further genetic and/or environmental risk factors may influence the age of onset (Table S2; see Supporting Information).^3^ Comparison within a family, despite the caveats associated with smaller numbers of patients, controls for some of this variation and is informative in our understanding of this disease. We also highlight the oral pustulation that was seen in our patient and that has been recognized by Navarini *et al*. as a useful indicator of *IL36RN* mutations.^7^

## Supporting information


**Table S1**. Familial homo‐ and heterozygous cases of generalized pustular psoriasis where at least two siblings share familial interleukin 36 receptor antagonist mutations.
**Table S2**. Cases of generalized pustular psoriasis with homozygous interleukin 36 receptor antagonist mutations, illustrating the range in age of onset seen across different populations.
