# Supplementary material for: A tale of two sisters: identical IL36RN mutations and discordant phenotypes
Source: Br J Dermatol. 2015 Nov 25;174(2):417–20. doi: 10.1111/bjd.14003 (PMC4762540; doi:10.1111/bjd.14003)
Supplement: Supplementary file 1 — Table S1. Familial homo‐ and heterozygous cases of generalized pustular psoriasis where at least two siblings share familial interleukin 36 receptor antagonist mutations. Table S2. Cases of generalized pustular psoriasis with homozygous interleukin 36 receptor antagonist mutations, illustrating the range in age of onset seen across different populations. [file BJD-174-417-s001.docx]

**“A tale of two sisters: identical *IL36RN* mutations and discordant phenotypes”**

*Neil Rajan^1^, Naomi Sinclair^1^, Hiroyuki Nakai^2^, Yutaka Shimomura^3^ and Sivakumar Natarajan^4^

^Supplementary file^

| **No.** | **Allele 1** | **Allele 2** | **Phenotype** | **Age of**  **Onset*** | **Affected**  **Siblings** | **Ethnicity** | **Pregnancy** | **Reference** |
| --- | --- | --- | --- | --- | --- | --- | --- | --- |
| 1 | c.28C>T/ p.Arg10X | c.115+6T>C/ p.Arg10ArgfsX | GPP | 2; 2 | 1 (twins) | Japanese | NR | ([Sugiura *et al.*, 2013](#_ENREF_11)) |
| 2 | c.338C>T/p.Ser113Leu | c.368C>T/ p.Thr123Met | GPP | 6 months; 34 years | 1 | British | Flare | Current publication |
| 3 | c.338C>T/p.Ser113Leu | c.338 C>T/ p.Ser113Leu | GPP 1 sib and ACH 2 sibs | 21; 30 | 3 | Lebanese | NR | ([Abbas *et al.*, 2013](#_ENREF_1)) |
| 4 | c.80T>C/ p.Leu27Pro | c.80T>C/ p.Leu27Pro | GPP | 2; 3 | 3 | Tunisian | NR | ([Marrakchi *et al.*, 2011](#_ENREF_5)) Family 1 |
| 5 | c.80T>C/ p.Leu27Pro | c.80T>C/ p.Leu27Pro | GPP | 1 week; 2 weeks; 11 years | 4 | Tunisian | NR | ([Marrakchi *et al.*, 2011](#_ENREF_5)) Family 2 |
| 6 | c.80T>C/ p.Leu27Pro | c.80T>C/ p.Leu27Pro | GPP | 2 weeks; 3 months; 6 years | 6 | Tunisian | Flare in female with age of onset at 3 months | ([Marrakchi *et al.*, 2011](#_ENREF_5)) Family 6 |

**Supplementary Table 1a. Familial homozygous and heterozygous cases of GPP, where at least 2 siblings share familial IL-36Ra mutations.**

Key: GPP – Generalised pustular psoriasis, PV – Psoriasis Vulgaris, ACH- Acrodermatitis Continuua of Hallopeau . Pink/Blue shading – heterozygous carriers, blue only shading – homozygous carriers.

* Ages of affected members shown in years, unless otherwise indicated.

| Homozygous Mutation | Age of onset | Origin | Reference |
| --- | --- | --- | --- |
| c.28C>T/ p.Arg10X | F: 47  M: 0.08  F: 65 | Japanese  Arabic  Japanese | ([Tominaga *et al.*, 2015](#_ENREF_12))  ([Renert-Yuval *et al.*, 2014](#_ENREF_8))  ([Sugiura *et al.*, 2013](#_ENREF_11)) |
| c.62 T>C/p.Leu21Pro | F: 1.5 | Pakistani | ([Ellingford *et al.*, 2015](#_ENREF_2)) |
| c.80T>C/ p.Leu27Pro | F: 1 - F: 35 | Tunisian | [(Marrakchi et al., 2011)](#RANGE!_ENREF_2) |
| c.95A>G/His32Arg | M: 14 | Iraqi | ([Körber *et al.*, 2013](#_ENREF_3)) |
| c.115+6T>C/ p.Arg10ArgfsX | F: 2 – F: 37  F: 0.17 – M: 53 | Malay  Chinese | ([Setta-Kaffetzi *et al.*, 2013](#_ENREF_9))  ([Li *et al.*, 2014](#_ENREF_4)) |
| c.227 C>T/Pro76Leu | M 0.33 | Turkish | ([Körber *et al.*, 2013](#_ENREF_3)) |
| c.338C>T/p.Ser113Leu | M: 5 - F: 51  F: 1  F: 17  M: 40 | European  Iraqi/German  German  British | ([Körber *et al.*, 2013](#_ENREF_3))  ([Onoufriadis *et al.*, 2011](#_ENREF_7))  ([Onoufriadis *et al.*, 2011](#_ENREF_7))  ([Navarini *et al.*, 2015](#_ENREF_6)) |
| c.368C>T/ p.Thr123Met | M: 20 | Japanese | ([Shiratori *et al.*, 2015](#_ENREF_10)) |

**Supplementary Table 1b. Cases of GPP with homozygous IL-36Ra mutations illustrating the range of ages of onset seen across different populations.**

Key: M= Male, F=Female.

* Ages of affected members shown in years.

**References (for Supplementary Table 1a and 1b)**

Abbas O, Itani S, Ghosn S, Kibbi AG, Fidawi G, Farooq M*, et al.* (2013) Acrodermatitis continua of Hallopeau is a clinical phenotype of DITRA: evidence that it is a variant of pustular psoriasis. *Dermatology (Basel, Switzerland)* 226:28-31.

Ellingford JM, Black GCM, Clayton TH, Judge M, Griffiths CEM, Warren RB (2015) A novel mutation in IL36RN underpins childhood pustular dermatosis. *Journal of the European Academy of Dermatology and Venereology*:n/a-n/a.

Körber A, Mössner R, Renner R, Sticht H, Wilsmann-Theis D, Schulz P*, et al.* (2013) Mutations in IL36RN in Patients with Generalized Pustular Psoriasis. *The Journal of investigative dermatology*:-.

Li X, Chen M, Fu Xa, Zhang Q, Wang Z, Yu G*, et al.* (2014) Mutation analysis of the IL36RN gene in Chinese patients with generalized pustular psoriasis with/without psoriasis vulgaris. *Journal of dermatological science*.

Marrakchi S, Guigue P, Renshaw BR, Puel A, Pei XY, Fraitag S*, et al.* (2011) Interleukin-36-receptor antagonist deficiency and generalized pustular psoriasis. *The New England journal of medicine* 365:620-8.

Navarini AA, Simpson MA, Borradori L, Yawalkar N, Schlapbach C (2015) Homozygous Missense Mutation in IL36RN in Generalized Pustular Dermatosis With Intraoral Involvement Compatible With Both AGEP and Generalized Pustular Psoriasis. *JAMA Dermatology* 151:452-3.

Onoufriadis A, Simpson MA, Pink AE, Di Meglio P, Smith CH, Pullabhatla V*, et al.* (2011) Mutations in IL36RN/IL1F5 Are Associated with the Severe Episodic Inflammatory Skin Disease Known as Generalized Pustular Psoriasis. *The American Journal of Human Genetics* 89:432-7.

Renert-Yuval Y, Horev L, Babay S, Tams S, Ramot Y, Zlotogorski A*, et al.* (2014) IL36RN mutation causing generalized pustular psoriasis in a Palestinian patient. *Int J Dermatol* 53:866-8.

Setta-Kaffetzi N, Navarini AA, Patel VM, Pullabhatla V, Pink AE, Choon S-E*, et al.* (2013) Rare Pathogenic Variants in IL36RN Underlie a Spectrum of Psoriasis-Associated Pustular Phenotypes. *Journal of Investigative Dermatology* 133:1366-9.

Shiratori T, Fukai K, Yasumizu M, Taguchi R, Tsuruta D, Abe Y*, et al.* (2015) IL36RNgene analysis of two Japanese patients with generalized pustular psoriasis. *International journal of dermatology* 54:e60-e2.

Sugiura K, Takemoto A, Yamaguchi M, Takahashi H, Shoda Y, Mitsuma T*, et al.* (2013) The majority of generalized pustular psoriasis without psoriasis vulgaris is caused by deficiency of interleukin-36 receptor antagonist. *J Invest Dermatol* 133:2514-21.

Tominaga C, Yamamoto M, Imai Y, Yamanishi K (2015) A Case of Old Age-Onset Generalized Pustular Psoriasis with a Deficiency of IL-36RN (DITRA) Treated by Granulocyte and Monocyte Apheresis. *Case Reports in Dermatology* 7:29-35.
